# Resistance exercise improves muscle strength, health status and pain intensity in fibromyalgia—a randomized controlled trial

**DOI:** 10.1186/s13075-015-0679-1

**Published:** 2015-06-18

**Authors:** Anette Larsson, Annie Palstam, Monika Löfgren, Malin Ernberg, Jan Bjersing, Indre Bileviciute-Ljungar, Björn Gerdle, Eva Kosek, Kaisa Mannerkorpi

**Affiliations:** Institute of Medicine, Department of Rheumatology and Inflammation research, Sahlgrenska Academy, University of Gothenburg, Guldhedsgatan Box 480, 405 30 Göteborg, Sweden; University of Gothenburg Centre for Person Centered Care (GPCC), Göteborg, Sweden; Department of Clinical Sciences, Danderyd Hospital, Karolinska Institute, Stockholm, Sweden; Department of Dental Medicine, Karolinska Institute, Stockholm, Sweden; Sahlgrenska University Hospital, Rheumatology, Göteborg, Sweden; Department of Pain and Rehabilitation Center, Linköping University, Linköping, Sweden; Department of Medical and Health Sciences, Linköping University, Linköping, Sweden; Department of Clinical Neuroscience, Karolinska Institute, Stockholm, Sweden; Institute of Neuroscience and Physiology, Section of Health and Rehabilitation, Physiotherapy, Sahlgrenska Academy, University of Gothenburg, Göteborg, Sweden

## Abstract

**Introduction:**

Fibromyalgia (FM) is characterized by persistent widespread pain, increased pain sensitivity and tenderness. Muscle strength in women with FM is reduced compared to healthy women. The aim of this study was to examine the effects of a progressive resistance exercise program on muscle strength, health status, and current pain intensity in women with FM.

**Methods:**

A total of 130 women with FM (age 22–64 years, symptom duration 0–35 years) were included in this assessor-blinded randomized controlled multi-center trial examining the effects of progressive resistance group exercise compared with an active control group. A person-centred model of exercise was used to support the participants’ self-confidence for management of exercise because of known risks of activity-induced pain in FM. The intervention was performed twice a week for 15 weeks and was supervised by experienced physiotherapists. Primary outcome measure was isometric knee-extension force (Steve Strong®), secondary outcome measures were health status (FIQ total score), current pain intensity (VAS), 6MWT, isometric elbow-flexion force, hand-grip force, health related quality of life, pain disability, pain acceptance, fear avoidance beliefs, and patient global impression of change (PGIC). Outcomes were assessed at baseline and immediately after the intervention. Long-term follow up comprised the self-reported questionnaires only and was conducted after 13–18 months. Between-group and within-group differences were calculated using non-parametric statistics.

**Results:**

Significant improvements were found for isometric knee-extension force (*p* = 0.010), health status (*p* = 0.038), current pain intensity (*p* = 0.033), 6MWT (*p* = 0.003), isometric elbow flexion force (*p* = 0.02), pain disability (*p* = 0.005), and pain acceptance (*p* = 0.043) in the resistance exercise group (*n* = 56) when compared to the control group (*n* = 49). PGIC differed significantly (*p* = 0.001) in favor of the resistance exercise group at post-treatment examinations. No significant differences between the resistance exercise group and the active control group were found regarding change in self-reported questionnaires from baseline to 13–18 months.

**Conclusions:**

Person-centered progressive resistance exercise was found to be a feasible mode of exercise for women with FM, improving muscle strength, health status, and current pain intensity when assessed immediately after the intervention.

**Trial registration:**

ClinicalTrials.gov identification number: NCT01226784, Oct 21, 2010.

## Introduction

Musculoskeletal pain has a negative impact on quality of life and work capacity [1] for individuals and entails large costs for society due to long-term sickness absence [2, 3]. Fibromyalgia (FM) affects approximately 1–3 % of the general population, and it is more common among women and in older age [4, 5]. FM is characterized by persistent widespread pain, increased pain sensitivity and tenderness [6] and is associated with impaired physical capacity [7–9], activity limitations [10], fatigue, and distress [6, 11].

The pain in FM is attributed to amplification of nociceptive input due to central sensitization and impaired central pain inhibition [12, 13]. Hypothetically, physical deconditioning leads to enhanced muscle ischemia, increasing peripheral sensitization and thus contributing to the central sensitization [14]. Although the precise etiology of FM is not known, physical deconditioning is believed to contribute to the development of FM [15]. Muscle strength in women with FM has been reported to be reduced by an average of 39 % compared to healthy women [16]. Possible physiological explanations for the reduced strength include structural changes in muscle fibers [17], altered neuromuscular control mechanisms [18], impaired blood circulation [19], and disturbances in regulation of growth and energy metabolism [20]. However, no differences in neuromuscular coordination was found in a study comparing exercising women with FM to sedentary controls, and both groups improved their motor unit activity during a resistance exercise program [21]. This indicates that a reason for reduced muscle strength in FM might be a low amount of physical activity at such a level that is required to maintain or improve muscle strength. Also a recent study using accelerometers indicates that the amount of physical activity at moderate and vigorous level is low among patients with FM when compared to healthy individuals [22]. Activity-induced pain is a common feature in FM [23] and might be a reason why the patients avoid activities and exercise, which could increase pain. Current guidelines for patients with FM include recommendations for aerobic exercise, such as brisk walking and cycling, as an important part of long-term management of FM [24, 25], as in several studies this mode of exercise has been shown to improve general health and physical function in patients with FM [26, 27]. Although muscle deconditioning is known to increase the susceptibility to microtrauma related to mechanical strain during physical activities [28], few studies have evaluated the effects of resistance exercise designed to improve muscle strength in FM [15]. However they have documented promising effects of resistance exercise on muscle strength, health status and pain, but the paucity of studies implies a low quality of evidence [15]. Meta-analyses in a Cochrane report of resistance exercise are based on one to three trials [15], warranting further research to improve confidence for estimated effects of resistance exercise for patients with FM [15].

A possible reason for the paucity of studies evaluating the effects of resistance exercise in FM is the risk of increased pain during isometric muscle contraction [23]. However exercise-induced pain during progressive resistance exercise might be avoided by gradual introduction to heavier loads [29]. Furthermore a theory of person-centeredness, which emphasizes active involvement of the patient in planning the treatment, is suggested to enhance the patient’s ability to manage health problems [30]. In the present study, the details of the exercise program were planned together with each patient, using the principles of person-centeredness, to support each participant’s ability to manage the exercise and the progress of it.

Relaxation therapy was chosen as an active control intervention as it is often integrated in multidisciplinary rehabilitation for patients with FM [24], rather than to control for treatment as usual. There is little evidence of the effects of relaxation therapy as an isolated therapy in FM [31], but relaxation therapy is assumed to improve overall wellbeing, thus providing a meaningful and inspiring therapy to control for the natural course and some aspects of attention and expectations. Patients recruited to the study were informed that the aim of the study was to compare these two treatment modalities. The aim of this study was to examine the effects of a progressive resistance exercise program using a person-centered approach, on muscle strength, health status, and current pain intensity in women with FM. Relaxation therapy was selected as an active control intervention.

## Methods

An assessor-blinded randomized controlled multicenter trial examined the effects of progressive resistance group exercise compared with an active control group. The trial was registered with ClinicalTrials.gov identification number: NCT01226784.

### Recruitment

The recruitment started in 2010 and data collection was completed at all sites (Gothenburg, Stockholm, and Linköping) in 2013. Inclusion criteria were women aged 20–65 years, meeting the American College of Rheumatology (ACR) 1990 classification criteria for FM [6]. Comorbidity as an exclusion criterion was defined by anamnesis. Exclusion criteria were high blood pressure (>160/90 mmHg), osteoarthritis (OA) in hip or knee, confirmed by radiological findings and affecting activities of daily life such as stair climbing or walking, other severe somatic or psychiatric disorders, other dominating causes of pain than FM, high consumption of alcohol (alcohol use disorders identification test (AUDIT) score >6) [32], participation in a rehabilitation program within the past year, regular resistance exercise or relaxation exercise twice a week or more, inability to understand or speak Swedish, and not being able to refrain from analgesics, non-steroidal anti-inflammatory drugs (NSAID) or hypnotic drugs for 48 hours prior to examinations.

**Fig. 1:**
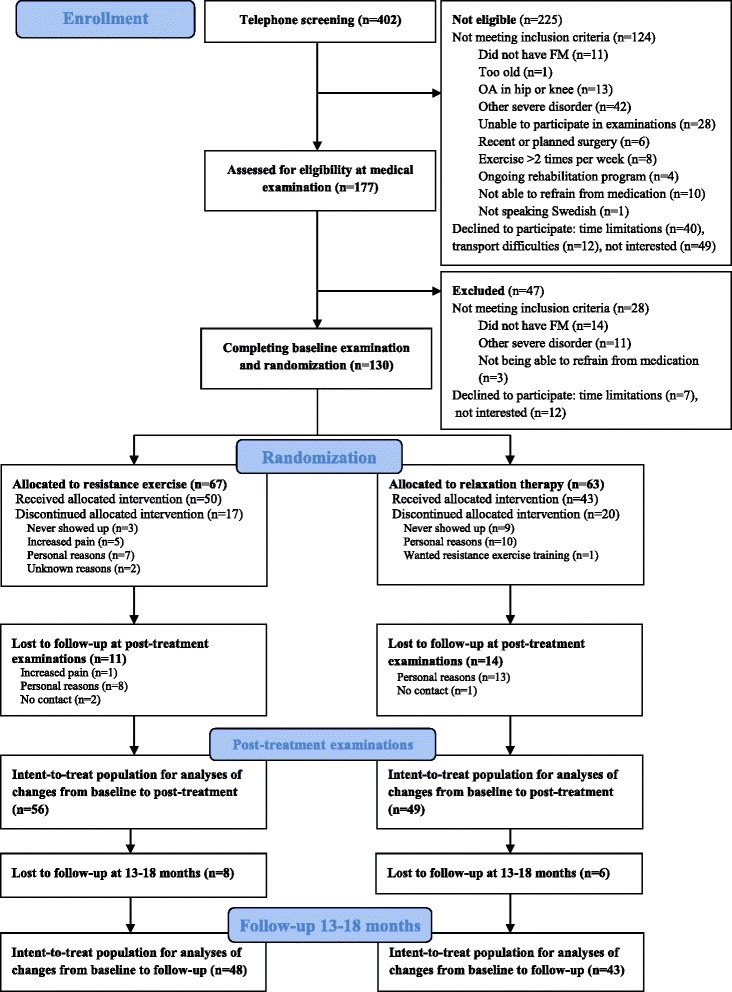
Consolidated Standards of Reporting Trials (CONSORT) flow diagram of the progress of the two groups of the randomized trial. *FM* fibromyalgia

Women with FM were recruited by newspaper advertisement in the local newspapers of three cities in Sweden (Gothenburg, Stockholm, and Linköping). A total of 402 women with FM who notified their interest for participation in the study were telephone screened for possible eligibility and informed about the study procedure. Out of these, 177 women who were interested in participation were referred for medical examination for further enrollment, while 225 were not eligible for enrollment (for details see Fig. 1). The 177 women were screened for eligibility by an experienced physician to verify ACR 1990 criteria for FM by means of a standardized interview and palpation of tender points [6]. A total of 47 women were found not eligible due to not meeting the inclusion criteria (n = 28), or declining participation (n = 19). One-hundred and thirty women with FM fulfilled the inclusion criteria. They were given written and oral information and were referred for baseline examinations (Fig. 1). Informed written consent was obtained from all participants before the baseline examination. After completing baseline examinations, the participants were randomized and informed of group allocation. An appointment for an individual introductory meeting with the specific physiotherapist guiding each intervention was scheduled with each participant. The study was approved for all sites by the Regional ethics committee in Stockholm (2010/1121-31/3).

### Randomization

Randomization was conducted separately for each site in blocks of six subjects by a computer generated sequence [33]. For each participant the treatment was concealed in sequentially numbered, sealed, opaque envelopes. Randomization and concealment was done by a person not involved in the examinations or treatments (ME). When a participant had been included the envelope was opened together with each participant, after which she was informed about the group to which she had been allocated.

### Intervention

Rationale for the resistance exercise program: the main goal was to improve muscle strength and health status by progressive resistance exercise, but without risking increased pain while loading the muscles. It was unclear how many participants would be able to manage exercise at higher loads. Exercise of large muscle groups, preferably muscles in the lower extremities were chosen, as risk of activity-induced pain was anticipated to be higher when loading muscles of upper extremities. Exercises improving core stability and power were included in the program.

Person-centered intervention: the resistance exercise program was performed twice a week for 15 weeks and was supervised by experienced physiotherapists. It was conducted at physiotherapy premises and at a local gym at four different sites in groups comprising five to seven participants to promote interaction between participants and to facilitate physiotherapeutic guidance. The intervention was preceded by an individual introductory meeting. The meeting was commenced with a dialogue between the participant and the physiotherapist about the participant’s earlier experiences and thoughts of exercise, which could potentially be an obstacle for her ability to exercise despite her explicit intention to do so. The introductory meeting also included exercise instructions, testing and adjustment of loads and modifications of specific exercises according to individual conditions and according to self-efficacy principles [34] of each participant’s confidence in their ability to perform each exercise and to manage specific loads. The meeting resulted in a written protocol with descriptions of specific exercises and loads, which was used by each participant as an exercise program at each exercise session. To promote the participant’s sense of control, and to avoid possible negative effects related to exercise, the exercise was initiated at low loads, and possibilities for progressions of loads were evaluated every 3−4 weeks in dialogue between the physiotherapist and participant (Box 1). When the participant was not ready to increase exercise loads, she continued exercising on the same loads until she was ready to do so. This mode of exercise was anticipated to increase exercise self-efficacy, enhance the ability to choose the proper level of exercise and better manage symptoms.

Estimation of one repetition maximum (1RM) was made by submaximal ratings of perceived exertion for health and safety reasons [35]. The participants were asked to perform their maximum number of repetitions until perceived exhaustion at an individually adjusted, given resistance. 1RM was based on the number of repetitions performed.

Active control group: the relaxation therapy was performed twice a week for 15 weeks and was guided by experienced physiotherapists. It was conducted at physiotherapy premises at four different sites in groups comprising five to eight participants and was preceded by an individual introductory meeting at the premises, which included instructions and allowed for preparations and modifications of practical matter such as positioning and the use of mattresses and pillows to reach a good level of comfort. The relaxation therapy was performed as autogenic training [31], which refers to a series of mental exercises including relaxation and autosuggestion. The physiotherapist guided the participants through their bodies, during approximately 25 minutes, by focusing their minds on the bodily experience of relaxation and letting the body part in focus rest on the ground. This was repeated for each specific body-part, aiming at feeling as relaxed as possible in the whole of the body at the end of the session. After each session the participants were invited to share experiences and ask each other and the physiotherapist questions, and continued thereafter with the stretching exercises.

### Assessment

Outcomes were assessed at baseline and at post-treatment examination after 15 weeks. Follow up was conducted 13–18 months after the baseline and only included self-reported questionnaires. All participants were invited to a post-treatment examination according to an intention-to-treat design. Baseline examinations and examinations after 15 weeks of intervention included serum samples for later analysis (not analyzed in this study), self-reported questionnaires, performance-based tests of muscle strength and physical capacity and assessment of current pain intensity. Background data were gathered using a standardized interview and included age, symptom duration, tender points, body mass index (BMI), level of leisure time physical activity (LTPAI) [36], pharmacological treatment, education, family status, country of birth, work status, and sick leave (Table 1). Examinations were conducted at physiotherapy premises by physiotherapists who were blinded to group allocation. Baseline and post-treatment examinations were performed by the same physiotherapists. The 13–18 month follow up comprised self-reported questionnaires only and was sent to the participants by mail. The participants who did not return the questionnaires in a reasonable time were reminded by telephone. After three reminders, participants that had not returned their questionnaires were regarded as missing. The participants that were already lost at post-treatment examinations were regarded as missing and were not contacted for follow up at 13–18 months.

**Table 1:** Characteristics of the study population

	Resistance exercise (experimental)	Relaxation therapy (control)	
	(n = 67)	(n = 63)	
	Mean (SD)	Mean (SD)	
Characteristics	Median (min; max)	Median (min; max)	p value
Age, years	50.81 (9.05)	52.10 (9.78)	0.27
	51 (25; 64)	55 (22; 64)	
Symptom duration, years	11.06 (8.53)	9.44 (7.33)	0.38
	9 (0; 35)	8 (0; 30)	
Tender points, number	15.76 (1.92)	15.49 (2.00)	0.48
	16 (11; 18)	16 (11; 18)	
Body mass index	27.39 (5.29)	28.66 (5.32)	0.06
	26 (20; 41)	27 (18; 43)	
LTPAI, h	5.62 (4.80)	5.79 (6.25)	0.52
	4 (0; 28)	4 (1; 38)	
	Number (%)	Number (%)	
Pharmacologigal treatment			
NSAID paracetamol			
Yes	53 (79 %)	44 (70 %)	0.22
Opioids for mild to moderate pain			
Yes	13 (19 %)	12 (19 %)	1.00
Antidepressants			
Yes	32 (48 %)	24 (38 %)	0.22
Anticonvulsives			
Yes	4 (6 %)	2 (3 %)	0.68
Sedatives			
Yes	11 (16 %)	12 (19 %)	0.82
Education			
≤9 years	8 (12 %)	15 (24 %)	
10–12 years	34 (51 %)	22 (35 %)	
>12 years	25 (37 %)	26 (41 %)	0.88
Living with an adult			
Yes	45 (67 %)	40 (64 %)	0.71
Born in Sweden	60 (90 %)	56 (89 %)	0.56
Work status			
0 %	29 (43 %)	26 (41 %)	
20–49 %	2 (3 %)	4 (6 %)	
50 %	13 (19 %)	11 (18 %)	
51–79 %	8 (12 %)	8 (13 %)	
100 %	15 (22 %)	14 (22 %)	0.71
Sick leave/disability pension			
25 %	9 (13 %)	5 (8 %)	
50 %	16 (24 %)	9 (14 %)	
75 %	2 (3 %)	3 (5 %)	
100 %	21 (31 %)	22 (35 %)	0.86

Missing values, LTPAI: *n* = 1. *NSAID* non-steroidal anti-inflammatory drugs, *LTPAI* leisure time physical activity instrument

### Outcomes

Resistance exercise was the core element of the intervention, but the program also involved other interacting components, such as the partnership between the physiotherapist and the participant when planning and progressing the exercise program. It was anticipated that both the voluntary activity of the participant and the severity of her health problems would influence the outcomes of the intervention. The prevalent evidence of the benefits of resistance exercise in FM is of low quality [15], and therefore muscle strength was selected as the primary outcome. Pain intensity, physical capacity, health status and other variables associated with health problems in chronic pain were selected for secondary outcomes, as exercising was also thought to impact on these variables.

The primary outcome was isometric knee-extension force (N) measured with a dynamometer (Steve Strong®: Stig Starke HBI, Göteborg, Sweden) using a standard protocol. The participant was in a fixed seated position with back support, knee and hip in 90 ° of flexion and legs hanging freely. A non-elastic strap was placed around the ankle and attached to a pressure transducer with an amplifier. The subjects were instructed and verbally encouraged to pull the ankle strap with maximal force for 5 seconds. Three trials were performed for each test and there was a one minute rest between each trial. The best performance out of three trials was recorded. A mean value from the right and left leg was calculated. The instrument has been used in previous studies of physical performance [37, 38] and has been reported to show satisfactory test-retest reliability for patients with a chronic condition [37].

Secondary outcomes were: the fibromyalgia impact questionnaire (FIQ) is a disease-specific self-reported questionnaire that comprises ten subscales of disabilities and symptoms ranging from 0 to 100. The total score is the mean of ten subscales. A higher score indicates a lower health status [39]. This instrument has shown good sensitivity in demonstrating therapeutic change [40]; current pain intensity (VAS), rated on a plastic 0-100 visual analogue scale with a moveable cursor along a line and anchors at the extremes. The participant was asked to rate her current pain intensity ranging from no pain at all to the worst imaginable pain; the six-minute walk test (6MWT), a performance-based test that measures total walking distance (m) during a period of 6 minutes [41]; maximal isometric elbow flexion force (kg) in both arms, was measured one by one using a dynamometer (Isobex®: Medical Device Solutions AG, Oberburg, Switzerland). The participant was in a seated position without back support, with the legs stretched out in front. The upper arm was aligned with the trunk and the elbow was placed in 90° of flexion. The maximum strength obtained during a period of 5 seconds was recorded and used in the present study [42]. Three trials were performed for each test and there was a one minute rest between each trial. The best performance out of three trials was recorded. A mean value from the right and left arm was calculated; hand grip force (N) bilaterally registered using Grippit® (AB Detektor, Göteborg, Sweden). The mean force over a set period of time (10 seconds) was recorded [43]. Two trials were performed for each test and there was a one minute rest between each trial. The best performance out of two trials was recorded. A mean value of the right and left hand was calculated and used in the present study; Short Form Health Survey (SF-36), a generic instrument assessing health related quality of life [44]. A higher score indicates better health. The subscales that build two composite scores, the physical component scale (PCS) and the mental component scale (MCS), were used in this study; the pain disability index (PDI), an instrument for measuring the impact that pain has on the ability of a person to participate in essential life activities on a scale from 0 to 70. The higher the index is, the greater the person’s disability due to pain [45, 46]; the chronic pain acceptance questionnaire (CPAQ), which assesses the degree of pain-related acceptance. It consists of 20 items ranging from 0 (never true) to 6 (always true). A higher score indicates a higher level of acceptance. The total score (0–120) is presented in this study [47]; the fear avoidance beliefs questionnaire (FABQ), a questionnaire with two sub-scales that assess the extent to which fear and avoidance affect work beliefs (7 items range 0–42) and physical beliefs (4 items 0–24) in patients with chronic pain. A higher score represents greater fear avoidance beliefs [48]; and the patient global impression of change (PGIC), a numeric scale ranging from 1 (very much improved) to 7 (very much worse), where a lower score indicates greater improvement. This instrument assesses perceived global impression of change from the patient’s perspective [49]. The PGIC was measured at post-treatment examinations and at 13–18 month follow up.

### Statistical analysis

Data were computerized and analyzed using the Statistical Package Software for the Social Sciences (SPSS version 22.0, Chicago, IL, USA). Descriptive data are presented as mean, SD, median (min; max) for continuous variables or the number (n) and percentage (%) for categorical variables. For comparison between two groups, the Mann-Whitney *U* test was used for continuous variables, Fisher’s exact test was used for dichotomous variables, and the Mantel Haenzel test was used for ordinal categorical variables. The Wilcoxon signed rank test was used for comparison between baseline and post-test within groups for continuous variables. Spearman correlation analysis was used for analyzing correlations between the PGIC and outcomes. To control possible type I errors, the upper limit of the expected number of false significant results for the analyses was calculated by the following formula:

$$ \alpha /1\hbox{--} \alpha \times \left(\mathrm{Number}\ \mathrm{of}\ \mathrm{tests}\ \hbox{--}\ \mathrm{Number}\ \mathrm{of}\ \mathrm{significant}\ \mathrm{tests}\right), $$

where α is the significance level. All significance tests were two-sided and conducted at the 5 % significance level. Outcomes were analyzed according to the intention-to-treat design, implying that all participants were invited to post-treatment examination, whether they had participated in the intervention or not. Only measured values were included in analyses of changes over time between the two groups and within the groups implying that cases missing were not included in the analysis. Effect size was calculated for variables showing a significant change. Effect size for between-group analyses was calculated by dividing the mean difference between the post-treatment score and baseline score in the intervention group and in the control group by the pooled SD for difference. Effect sizes from 0.20 to <0.50 were regarded as small, while effect sizes from 0.50 to <0.80 were regarded as moderate [50]. No previous data were found for isometric knee-extension force in FM using the selected dynamometer (the primary outcome), but the same methodology was applied in a study of women with chronic disease. Their isometric knee-extension force was 263 N, SD 100 [37]. Based on that report, 59 participants per group would be satisfactory to detect a 20 % difference with 80 % power when the significance level was set to 5 %.

## Results

### Participants

There were no significant baseline differences between the resistance exercise group (n = 67) and the active control group (n = 63) in background data (Table 1), or the primary outcome or secondary outcomes.

#### Intention-to-treat analysis

All participants were invited to a post-treatment examination according to the intent-to-treat design and 81 % of the total sample completed the test, 56 (84 %) belonging to the resistance exercise group and 49 (76 %) in the active control group (Fig. 1). A total of 17 participants (25 %) in the resistance exercise group, and 20 (32 %) in the active control group discontinued the intervention for various reasons (Fig. 1). No significant differences were found when comparing the baseline characteristics of the women who completed and the women who failed to complete the post-treatment examinations. Adverse effects were reported by five participants, all in the resistance exercise group, who chose to discontinue the intervention due to increased pain (Fig. 1), but two of these participants completed post-treatment examinations. Mean attendance rate at the resistance exercise sessions was 71 % and 64 % at the relaxation therapy sessions (range 0 to 100 % in both groups).

#### Exercise loads

A total of 42 participants (62.7 %) in the resistance exercise group reached exercise loads of 80 % of 1RM while 7 participants (10.4 %) reached exercise loads of 60 % of 1RM. The women in the resistance exercise group who managed to reach exercise loads of 80 % of 1RM (n = 42, 63 %) showed significantly better physical capacity represented by 6MWT (*p* = 0.040) and health status represented by FIQ total score (*p* = 0.029) at baseline than the women in the resistance exercise group who did not reach exercise levels of 80 % of 1RM (Table 2).

**Table 2:** Comparison of baseline values for participants in the resistance exercise group who reached exercise loads of 80 % and participants in the resistance exercise group who reached exercise loads up to 60 %

	Resistance exercise (experimental)		
	Baseline values of participants reaching loads of 80 %	Baseline values of participants reaching loads ≤60 %	Comparison of baseline values between groups
	(n = 42)	(n = 25)	
Measures	Mean (SD)	Mean (SD)	*P* value
	Median (min; max)	Median (min; max)	
Age, years	50.26 (9.65)	51.7 (8.1)	0.68
	51 (25; 64)	52 (33; 63)	
Symptom duration, years	11.44 (8.31)	10.4 (9.0)	0.47
	10 (0; 35)	8 (0; 30)	
Tender point count, number	15.57 (1.92)	16.08 (1.91)	0.30
	16 (11; 18)	17 (12; 18)	
Body mass index	26.75 (4.2)	28.5 (6.7)	0.48
	26 (21; 36)	27 (20; 41)	
LTPAI, h	4.87 (3.87)	6.9 (5.9)	0.17
	4 (0;18)	6 (1;28)	
Primary outcome			
Isometric knee-extension force, N	347.3 (106.2)	301.2 (110.7)	0.11
	344 (114;643)	305 (111;585)	
Secondary outcomes			
FIQ total score (0-100)	57.3 (12.7)	65.9 (15.8)	**0.029**
	55.2 (31; 81)	64 (40; 95)	
Current pain intensity, visual analog scale	49.5 (22.1)	48.8 (27.19)	0.68
	54 (12; 89)	50 (5;100)	
6MWT, m	573.7 (70.3)	527.7 (75.5)	**0.040**
	570 (376; 766)	548 (360; 655)	
Isometric elbow-flexion force, kg	13.1 (5.3)	12.9 (5.7)	0.76
	13 (2; 26)	13 (3; 32)	
Isometric hand-grip force, N	168.4 (70.4)	150.7 (65.6)	0.30
	162 (34; 319)	169 (62; 315)	
SF36 PCS, 0–100	31.7 (7.5)	30.3 (8.8)	0.32
	31 (12; 49)	30 (18; 49)	
SF36 MCS, 0–100	39.8 (11.9)	34.0 (12.1)	0.10
	41 (10; 59)	35 (13; 61)	
PDI, 0–70	34.3 (11.6)	36.9 (13.4)	0.40
	34 (12; 69)	39 (8; 62)	
CPAQ total, 0–120	66.0 (14.5)	59.5 (18.0)	0.13
	64 (35; 99)	59 (19; 106)	
FABQ_physical_, 0–24	9.0 (5.9)	12.6 (7.1)	0.23
	9 (0; 23)	13 (2; 24)	
FABQ_work_, 0–42	16.1 (12.3)	20.0 (13.9)	0.37
	14 (0; 42)	21 (0; 39)	

*6MWT* 6 minute walk test, *SF36 PCS* Short Form 36 physical component scale, *SF36 MCS* Short Form 36 mental component scale, *CPAQ* chronic pain acceptance questionnaire, *PDI* pain disability index, *FABQ* fear avoidance beliefs questionnaire

Type I error: the between-group analyses comprised a total of 12 statistical analyses, with 7 significant values at the significance level 0.05, and the upper level of the number of false significant results was 0.26, which indicates that 0–1 of the significant results observed might be false.

### Primary outcome

Significantly greater improvement (*p* = 0.010) was found for isometric knee-extension force in favor of the resistance exercise group as compared to the active control group (Table 3). The effect size of change in isometric knee-extension force for the intervention group compared with the active control group was 0.55 (i.e., a moderate effect size). A total of 30 % of the participants fulfilling the resistance exercise program (n = 49) improved their isometric knee-extension force by 20 % or more, while the changes ranged from 51 % to 126 % on the individual level, implying large variation among the participants.

**Table 3:** Between-group analysis and within-group analysis of the primary and secondary outcomes

Measures	Resistance exercise (experimental)				Relaxation therapy (control)				Between-group analysis of change
	Baseline	Post test	Post-test- baseline	Within-group analysis	Baseline	Post test	Post-test- baseline	Within-group analysis	
	(n = 67)	(n = 56)	(n = 56)		(n = 63)	(n = 49)	(n = 49)		
	Mean (SD)	Mean (SD)	Δ (SD)	*p* value	Mean (SD)	Mean (SD)	Δ (SD)	*p* value	*p* value
	Median (min; max)	Median (min; max)	Δ (min; max)		Median (min; max)	Median (min; max)	Δ (min; max)		
Primary outcome									
Isometric knee-extension force, N	330.1 (109.4)	356.2 (118.9)	30.4 (71.9)	**0.002**	298.8 (107.8)	276.4 (112.9)	−8.8 (70.0)	0.644	**0.010**
	326 (111; 643)	342 (105; 663)	31 (−157; 178)		287 (51; 534)	278 (41; 595)	−10 (−222; 132)		
*Secondary outcomes*									
FIQ total, 0–100	60.5 (14.4)	54.4 (18.2)	−5.7 (15.0)	**0.009**	61.1 (17.3)	59.3 (16.0)	0.1 (12.9)	0.71	**0.038**
	59 (31; 95)	55 (11; 88)	−3 (−51; 20)		61 (17; 88)	61 (21; 86)	1 (−34; 25)		
Current pain intensity, VAS	49.3 (23.9)	38.6 (25.2)	−11.5 (25.1)	**0.002**	52.4 (18.3)	53.4 (20.0)	−1.5 (16.5)	0.63	**0.033**
	50 (5; 100)	31 (0; 95)	−13 (−83; 48)		51 (10; 88)	56 (10; 86)	−2 (−51; 30)		
6MWT, m	556.6 (75.1)	579.7 (73.7)	18.4 (65.1)	**0.002**	540.7 (64.5)	533.9 (73.1)	−5.6 (43.5)	0.51	**0.003**
	566 (360; 766)	582 (340; 762)	24 (−290; 196)		530 (362; 660)	537 (366; 656)	1 (−125; 101)		
Isometric elbow flexion force, kg	13.0 (5.4)	14.8 (5.6)	2.4 (3.3)	**<0.001**	10.9 (5.2)	11.7 (5.6)	1.2 (3.3)	**0.020**	**0.020**
	13 (2; 32)	15 (2; 27)	2 (−5; 12)		10 (2; 24)	12 (3; 27)	1 (−7; 13)		
Hand-grip force, N	161.8 (68.7)	181.1 (61.5)	20.1 (36.1)	**<0.001**	139.4 (61.7)	147.2 (66.7)	14.0 (37.9)	**0.013**	0.49
	164 (34; 319)	185 (38; 327)	14 (−32; 158)		134 (40; 311)	146 (39; 327)	9 (−101; 98)		
SF36 PCS, 0–100	31.2 (7.9)	34.5 (9.1)	3.3 (7.2)	**0.004**	29.9 (8.1)	30.7 (8.3)	0.8 (5.7)	0.28	0.11
	31 (12; 50)	35 (14; 54)	3 (−13; 18)		30 (10; 50)	30 (17; 47)	1 (−13; 13)		
SF36 MCS, 0–100	37.7 (12.2)	42.0 (12.6)	3.3 (10.3)	**0.007**	39.6 (12.1)	38.8 (12.9)	−0.4 (9.5)	0.86	0.054
	37 (10; 61)	44 (12; 62)	3 (−23; 35)		42 (16; 59)	41 (13; 61)	0 (−22; 23)		
PDI, 0–70	35.3 (12.2)	32.2 (13.1)	−3.8 (10.6)	**0.006**	35.0 (12.5)	35.7 (12.4)	1.4 (9.0)	0.27	**0.005**
	36 (8; 69)	34 (7; 67)	−5 (−29; 23)		34 (7; 61)	38 (9; 58)	0 (−19; 21)		
CPAQ, 0–120	63.6 (16.1)	69.6 (15.2)	5.7 (13.1)	**0.002**	62.4 (17.1)	63.4 (19.1)	0.1 (11.8)	0.79	**0.043**
	63 (19; 106)	69 (34; 98)	6 (−27; 46)		61 (15; 107)	60 (30; 113)	2 (−38; 23)		
FABQ_physical_, 0–24	9.7 (6.1)	8.9 (6.1)	−0.8 (7.0)	0.36	11.2 (6.1)	10.3 (6.3)	−1.3 (5.6)	0.24	0.92
	9 (0; 24)	8 (0; 22)	−1 (−19;19)		11 (0; 24)	9 (0; 24)	0 (−18;10)		
FABQ_work_, 0–42	17.2 (12.7)	17.8 (13.1)	0.4 (8.9)	0.83	15.9 (12.1)	16.67 (12.5)	1.2 (8.1)	0.54	0.79
	16 (0; 42)	16 (0; 42)	0 (−27; 29)		12 (0; 42)	14 (0; 42)	0 (−19; 30)		

Missing values at baseline: Resistance exercise group, SF36 PCS and SF36 MCS, n = 1; FABQ_work_, n = 6, FABQ_physical_, n = 1; Relaxation therapy group, CPAQ, n = 1; FABQ_work_, n = 8. Missing post-test values: Resistance exercise group, SF36 MCS and PCS, n = 3; FABQ_work_, n = 7; FABQ_physical_, n = 2; Relaxation therapy group, FIQtotal, n = 1; SF36 PCS and MCS, n = 2; FABQ_work_, n = 9. Significant *p* values are shown in bold text. *6MWT* six-minute walk test, *FIQ* fibromyalgia impact questionnaire, *VAS* visual analog scale, *SF36* short-form 36, *PDI* pain disability index, *CPAQ* chronic pain acceptance questionnaire, *FABQ* fear avoidance beliefs questionnaire

No significant baseline differences (*p* = 0.51) were found for isometric knee-extension force between the three sites. Mean difference for change in isometric knee-extension force from baseline to post-treatment examinations between the two groups was 43.0 N (standard error (SE) 22.5), 40.1 N (SE 24.6), and 35.2 N (SE 23.1) at each site respectively.

### Secondary outcomes

Significantly greater improvement was observed in health status (FIQ total score) (*p* = 0.038) in the resistance exercise group compared to the active control group (Table 3). The effect size of the change in the FIQ total score for the intervention group compared with the active control group was 0.41. Significantly greater improvement was observed in current pain intensity (VAS) (*p* = 0.033) in the resistance exercise group compared to the active control group (Table 3). The effect size of the change in current pain intensity for the intervention group compared with the active control group was 0.46. Significantly greater improvement was observed in the 6MWT (*p* = 0.003) in the resistance exercise group compared to the active control group (Table 3). The effect size of the change in the 6MWT for the intervention group compared with the active control group was 0.45. Significantly greater improvement was observed in isometric elbow-flexion force (*p* = 0.020) in the resistance exercise group compared with the active control group (Table 3). The effect size of the change in isometric elbow flexion force for the intervention group compared with the active control group was 0.36. There was no significant difference between groups in hand-grip force; both the exercise intervention group and the active control group improved their strength significantly (*p* <0.001, *p* = 0.013) (Table 3).

Significant improvements were observed in the health related quality of life (SF-36 PCS and MCS) (*p* = 0.007) within the resistance exercise group, reflecting what is considered to be a clinically important difference [51]. No significant differences were found when comparing the resistance exercise group with the active control group (Table 3). Significantly greater improvement in pain disability represented by the PDI (*p* = 0.005) was observed in the resistance exercise group compared to the active control group (Table 3). The effect size of the change in the PDI for the intervention group compared with the active control group was 0.53. Significantly greater improvement in pain acceptance represented by the CPAQ (*p* = 0.043) was observed in the resistance exercise group as compared to the active control group (Table 3). The effect size of the change in the CPAQ for the intervention group compared with the active control group was 0.45. No differences within groups or between groups were found for fear avoidance beliefs represented by the FABQ (Table 3).

### PGIC

PGIC differed significantly (*p* = 0.001) in favor of the resistance exercise group as compared with the active control group at post-treatment examinations. A total of 62.5 % of the participants in the resistance exercise group and 32.7 % in the active control group reported improvement in symptoms. PGIC ratings correlated significantly with improvements in current pain intensity (VAS) (*r*_s_ 0.38, *p* = 0.004) and SF-36 PCS (*r*_s_ 0.54, *p* <0.001) in the resistance exercise group.

There were no significant differences in the level of leisure time physical activity (LTPAI) (*p* = 0.74) between the resistance exercise group (5.6 h, SD 5.1) and the active control group (5.9 h, SD 5.2) at baseline. The level of moderate to vigorous physical activity at baseline in the resistance exercise group was 2.4 h (SD 2.6) and in the active control group 2.2 h (SD 2.1) (*p* = 0.89). During the intervention period, the level of physical activity increased significantly (*p* <0.001) in the resistance exercise group (2.3 h, SD 4.8) compared to the active control group (−0.1 h, SD 4.8). Moderate to vigorous physical activity increased significantly more (*p* = 0.003) in the resistance exercise group (1.8 h, SD 3.0) compared to the active control group (0.4 h, SD 2.6).

### Follow up at 13–18 months

A total of 91 (70 %) participants completed the follow up, 48 (72 %) in the resistance exercise group and 43 (68 %) in the active control group, respectively (Fig. 1). No significant differences between the resistance exercise group and the active control group were found at follow up after 13–18 months when compared to baseline measures of these outcomes (Table 4). The only significant within-group improvement at follow up in the resistance exercise group was for pain acceptance (CPAQ) (*p* = 0.044) (Table 4).

**Table 4:** Between-group analysis and within-group analysis of outcomes assessed at follow up after 13–18 months

Measures	Resistance exercise (experimental)				Relaxation therapy (control)				Between group analysis for change
	Baseline	13–18 month follow up	Follow up- baseline	Within-group analysis	Baseline	13–18 month follow up	Follow up-baseline	Within-group analysis	
	(n = 67)	(n = 48)	(n = 48)		(n = 63)	(n = 43)	(n = 43)		
	Mean (SD)	Mean (SD)	Δ (SD)	*p* value	Mean (SD)	Mean (SD)	Δ (SD)	*p* value	*p* value
	Median (min; max)	Median (min; max)	Δ (min; max)		Median (min; max)	Median (min; max)	Δ (min; max)		
Secondary outcomes									
FIQ total, 0–100	60.5 (14.4)	57.1 (19.4)	−1.8 (17.0)	0.71	61.1 (17.3)	55.4 (17.0)	−3.4 (12.3)	0.065	0.35
	59 (31; 95)	59 (17; 92)	1 (−45; 35)		61 (17; 88)	55 (15; 90)	−3 (−28; 36)		
Current pain intensity, VAS	49.3 (23.9)	49.2 (20.8)	0.7 (23.4)	0.87	52.4 (18.3)	52.1 (19.5)	−2.9 (19.6)	0.45	0.52
	50 (5; 100)	47 (9; 84)	3 (−73; 58)		51 (10; 88)	53 (5; 83)	−2 (−62; 46)		
SF36 PCS, 0–100	31.2 (7.9)	32.2 (8.0)	0.8 (7.1)	0.39	29.9 (8.1)	32.0 (9.4)	1.9 (7.3)	0.16	0.62
	31 (12; 50)	30 (17; 54)	1 (−14; 14)		30 (10; 49)	32 (17; 52)	1 (−13; 19)		
SF36 MCS, 0–100	37.7 (12.2)	39.2 (13.9)	−0.4 (13.5)	0.78	39.6 (12.1)	40.0 (11.9)	−0.4 (12.2)	0.66	0.89
	37 (10; 61)	39 (10; 59)	0 (−28; 29)		42 (16; 59)	43 (19; 60)	−2 (−23; 28)		
PDI, 0–70	35.3 (12.2)	33.0 (11.6)	−2.6 (9.7)	0.094	35.0 (12.5)	33.7 (10.9)	−0.3 (10.1)	0.58	0.42
	36 (8; 69)	34 (7; 62)	−3 (−28; 17)		34 (7; 61)	35 (8; 54)	−1 (−21; 25)		
CPAQ total, 0–120	63.6 (16.1)	68.0 (15.4)	3.6 (11.3)	**0.044**	62.4 (17.1)	66.6 (17.1)	2.3 (14.0)	0.21	0.76
	63 (19; 106)	70 (33; 100)	3 (−21; 32)		61 (15; 107)	63 (24; 101)	1 (−35; 28)		
FABQ_physical_, 0–24	9.7 (6.1)	10.1 (5.2)	−0.1 (5.5)	0.57	11.2 (6.1)	9.7 (6.3)	−1.6 (4.5)	0.051	0.38
	9 (0; 24)	10 (2; 21)	0 (−10; 17)		11 (0; 24)	10 (0; 24)	−1 (−14; 5)		
FABQ_work_, 0–42	17.2 (12.7)	15.5 (11.5)	−1.8 (9.7)	0.20	15.9 (12.1)	14.7 (12.4)	−1.0 (11.2)	0.66	0.59
	16 (0; 42)	14 (0; 41)	−1 (−30; 32)		12 (0; 42)	13 (0; 42)	0 (−41; 30)		

Missing values at baseline: Resistance exercise group, SF36 PCS and SF36 MCS, n = 1; FABQ_work_, n = 6; FABQ_physical_, n = 1. Relaxation therapy group, CPAQ, n = 1; FABQ_work_, n = 8. Missing values at 13–18 month follow up, Resistance exercise group, current pain intensity, n = 2; FABQ_work_, n = 5; SF36 PCS and SF36 MCS, n = 1. Relaxation therapy group, FABQ_work_, n = 7; FABQ_physical_, n = 1; SF36 PCS and SF36 MCS, n = 1. Significant *p* values are shown in bold text. *FIQ* fibromyalgia impact questionnaire, *VAS* visual analog scale, *SF36* short-form 36, *PDI* pain disability index, *CPAQ* chronic pain acceptance questionnaire, *FABQ* fear avoidance beliefs questionnaire

There was no significant difference (*p* = 0.07) in the level of leisure time physical activity (LTPAI) from baseline to follow up between the resistance exercise group (−0.4, SD 4.9) and the active control group (−1.4, SD 2.6), as both groups had slightly decreased their total physical activity. However, at the same time, both groups had increased the level of moderate to vigorous physical activity (*p* = 0.74), represented by 0.8 h (SD 4.5) in the resistance exercise group and 0.9 h (SD 3.1) in the active control group.

## Discussion

The main findings of this study were significant improvements in isometric knee-extension force, current pain intensity, and other aspects of health in the resistance exercise group compared to the active control group. These results were supported by significant within-group improvements in the resistance exercise group. The improvement of the knee-extension force is in line with a previous study of resistance exercise in women with FM [21]. The effect size of change in the isometric knee-extension force indicated a moderate improvement in the resistance exercise group as compared with the control group. The mean improvement in isometric knee-extension force was smaller in our sample than in the two previous studies [21, 52], and one reason for this might be the longer intervention period of these studies which was 21 weeks compared to 15 weeks in our study [21, 52]. Other reasons for the difference in improvement may be related to differences in the measurement equipment, population characteristics, disease severity and exercise parameters. The significant between-group differences found in elbow-flexion force in favor of the resistance exercise group were supported by significant within-group improvements in the resistance exercise group. To our knowledge this is the first resistance exercise study showing that women with FM can improve their biceps strength by resistance exercise. Elbow-flexion force and hand-grip force had also improved significantly in the active control group. A reason for the improvement might be reduced tension in upper-extremity muscles. A previous study showed that patients with FM displayed higher levels of unnecessary tension in shoulder flexors and also had reduced strength during dynamic activities compared to pain-free controls [53]. Our interpretation is that the relaxation therapy resulted in lower tension in the shoulder muscles and thus increased the hand-grip force. Also, walking capacity, measured with 6MWT improved with resistance exercise which is in line with previous reports of resistance exercise in women with FM [54].

The mean improvement in current pain intensity in the resistance exercise group represented an improvement of 23 %, which is considered a clinically important difference, as a reduction of 15 % represents a minimal clinically important difference [55]. The improvements in pain intensity are in line with reports from previous studies of improvements in pain following resistance exercise in FM [21, 54, 56, 57]. Also, the improvements in FIQ total score support previous findings in studies of women with FM engaging in resistance exercise [56, 57].

PGIC differed significantly in favor of the resistance exercise group. We found that PGIC correlated with improvements in current pain intensity and SF-36 physical component score, which implies that the participants’ overall impressions of change reflect clinically important improvements in disease-related health problems. These findings are in line with a previous report on FM from OMERACT [58]. Notable is that the improvements in SF36 scores in the resistance exercise group can be regarded as clinically important differences [51]. Significantly improved pain disability (assessed by the PDI) with a moderate effect-size for change indicated improvement in participation in everyday life activities, which reflects that the intervention focusing on enhancement in self-confidence and pain management during the exercise sessions was successful. Further, significantly improved pain acceptance (assessed by the CPAQ), was found in the resistance exercise group compared with the active control group. Acceptance of pain is assumed to be associated with less disability and better functioning in patients with chronic pain [47], and the results of this study indicate that pain acceptance can be improved when engaging in exercise.

The mean attendance rate was 71 % at the resistance exercise sessions and 64 % at the relaxation therapy sessions, which is regarded as a satisfactory rate in patients with severe health problems. The progression of the resistance exercise program proved to be a successful mode of exercise for most participants, as the majority tolerated the exercise well and few participants experienced aggravated symptoms.

Pain acceptance (CPAQ) was the only significant improvement found at follow up after 13–18 months in the resistance exercise group, which implies that the intervention promoted a process of pain acceptance that has long-term effects. However this finding should be interpreted with caution due to the fact that multiple comparisons were conducted. A probable reason for the lack of other long-term effects is that physical activity levels declined to baseline levels after the end of the intervention period. This implies that the participants had difficulties with maintaining regular resistance exercise without supervision. Some of the reasons given by participants for not continuing exercising were expensive gym membership, need for continued supervision and guidance, and difficulties in prioritizing exercise in daily life. A similar lack of long-lasting effects and difficulties among women with FM to maintain their levels of resistance exercise after the end of intervention have previously been reported [57]. A longer period of guidance and support might increase the prospects for long-lasting effects [59].

In this study, 81 % of the study population completed post-treatment examination, indicating satisfactory compliance. Five participants (7 %) in the resistance exercise group reported adverse effects and three of these did not complete the post-treatment examination. Adverse effects in this study are in line with a previous study of resistance exercise in women with FM [56]. In this study, 63 % of the participants managed to attain exercise loads of 80 % of 1RM. At baseline, these participants presented with better physical capacity in terms of 6MWT, and health status in terms of FIQ total score than those who did not reach loads of 80 %, implying that personal instructions and progression of exercise loads need to be adjusted to the participants’ physical resources and health status.

Muscle-strengthening activity, such as resistance exercise, at least twice a week is recommended for preventing age-related loss of muscle mass, impaired physical function [60] and the development of degenerative age-related chronic conditions [61] in older adults in the general population. The prevention of loss of muscle mass and physical function due to aging could be argued as even more important in this population given the impaired muscle strength [8, 9] and reduced levels of physical activity [22] previously shown in women with FM. Further, the benefits of regular progressive resistance exercise on muscle strength, pain, health status and participation in daily life activities shown in this study implies that resistance exercise can be recommended as a safe and effective option for exercise, which warrants inclusion in the management of FM.

### Implications

The positive results of the study showed that a supervised resistance exercise program based on person-centered principles with individually adjusted loads and progression according to each participant’s resources is feasible and successful. This program can be recommended to the general population of women with FM as the characteristics of our study sample in terms of tender points, pain intensity levels and FIQ scores appear to be representative of women with FM in general [54, 56, 62]. However, strategies to support long-term regular exercise should be developed to ensure longstanding health effects.

### Limitations

The recruitment procedure i.e., newspaper advertisement may have resulted in recruitment of participants who were motivated to exercise and this could bias the results. To minimize this risk the advert was designed to recruit participants to both interventions so none of the participants would know in advance which intervention was the active intervention or the control intervention. Unlike pharmacological studies, which are easily blinded, behavioral and physical treatment requiring the active participation of patients is virtually impossible to blind or make inert.

## Conclusion

Person-centered progressive resistance exercise was shown to be a feasible mode of exercise for women with FM, improving muscle function, health status, current pain intensity, pain management and participation in activities of daily life. At long-term follow up the effects had declined to baseline levels, implying that a longer period of guidance and support is recommended to increase the possibilities of maintaining regular exercise habits.

## Box 1 Dosage of resistance exercise, individualized according to each participant’s resources

*Frequency:* 2 times per week for 15 weeks, supervised by physiotherapists

*Exercise sessions:* 10 minute warm up, 50 minute standardized protocol including: leg-press, knee-extension and knee-flexion using weight machine, biceps curl and hand grip strength using free weights, heel raise and core stability using body weight and 10 minutes of stretching exercises. Exercises for explosive strength were added to the protocol five weeks, and eight weeks into the intervention with rapid heel-raises and explosive knee-extensions respectively.

*Progression:* baseline: 40 % of 1 RM, with 15–20 repetitions in 1–2 sets. Three to four weeks: 60 % of 1 RM, with 10–12 repetitions in 1–2 sets. 6–8 weeks: 80 % of 1RM, performed with 5–8 repetitions in 1–2 sets.

Between each set there was a 1 minute recovery.

## Abbreviations

1RM: one repetition maximum
6MWT: six minute walk test
ACR: American College of Rheumatology
BMI: body mass index
CPAQ: chronic pain acceptance questionnaire
FIQ: fibromyalgia Impact Questionnaire
FM: fibromyalgia
LTPAI: leisure time physical activity
NSAID: non-steroidal anti-inflammatory drugs
PGIC: patient global impression of change
SE: standard error
SF36: Short Form 36
SF36-MCS: Short Form 36-mental component scale
SF36-PCS: Short Form 36-physical component scale
VAS: visual analog scale
